# Tandem gene duplication selected by activation of horizontally transferred gene in bacteria

**DOI:** 10.1007/s00253-024-13160-z

**Published:** 2024-05-23

**Authors:** Fangqing Zhang, Xinxin Shi, Jian Xu, Wen Yuan, Zhichao Li

**Affiliations:** 1 Key Laboratory of Systems Microbial Biotechnology, Tianjin Institute of Industrial Biotechnology, Chinese Academy of Sciences, Tianjin, 300308 China; 2 National Technology Innovation Center of Synthetic Biology, Tianjin, 300308 China; 3 Key Lab of Industrial Fermentation Microbiology of the Ministry of Education, School of Biotechnology, Tianjin University of Science and Technology, Tianjin, China

**Keywords:** Horizontal gene transfer, Tandem gene duplication, Gene expression regulation, Industrial bacterium

## Abstract

**Abstract:**

Horizontal gene transfer occurs frequently in bacteria, but the mechanism driving activation and optimization of the expression of horizontally transferred genes (HTGs) in new recipient strains is not clear. Our previous study found that spontaneous tandem DNA duplication resulted in rapid activation of HTGs. Here, we took advantage of this finding to develop a novel technique for tandem gene duplication, named tandem gene duplication selected by activation of horizontally transferred gene in bacteria (TDAH), in which tandem duplication was selected by the activation of horizontally transferred selectable marker gene. TDAH construction does not contain any reported functional elements based on homologous or site-specific recombination and DNA amplification. TDAH only contains an essential selectable marker for copy number selection and 9-bp-microhomology border sequences for precise illegitimate recombination. One transformation and 3 days were enough to produce a high-copy strain, so its procedure is simple and fast. Without subsequent knockout of the endogenous recombination system, TDAH could also generate the relatively stable high-copy tandem duplication for plasmid-carried and genome-integrated DNA. TDAH also showed an excellent capacity for increase gene expression and worked well in different industrial bacteria. We also applied TDAH to select the optimal high copy number of *ribA* for vitamin B_2_ production in *E. coli*; the yield was improved by 3.5 times and remained stable even after 12 subcultures. TDAH is a useful tool for recombinant protein production and expression optimization of biosynthetic pathways.

**Key points:**

*• We develop a novel and efficient technique (TDAH) for tandem gene duplication in bacterium. TDAH is based on the mechanism of HTG rapid activation. TDAH does not contain any reported functional elements based on homologous recombination and DNA amplification. TDAH only contains an essential selectable marker for copy number selection, so its construction and procedure are very simple and fast.*

*• TDAH is the first reported selected and stable tandem-gene-duplication technique in which the selected high-copy plasmid-carried and genome-integrated DNA could remain stable without the subsequent knockout of recombination system.*

*• TDAH showed an excellent capacity for regulating gene expression and worked well in different industrial bacteria, indicating it is a useful tool for recombinant protein production and expression optimization of biosynthetic pathways.*

*• TDAH was applied to select the optimal high copy number of ribA for vitamin B*_*2*_ *production in E. coli; the yield was improved by 3.5-fold and remained stable even after 12 subcultures.*

**Supplementary Information:**

The online version contains supplementary material available at 10.1007/s00253-024-13160-z.

## Introduction

Horizontal gene transfer (HGT), also known as lateral gene transfer (LGT), is the exchange of genetic information between unrelated organisms, which stands in contrast to vertical gene transfer (VGT) from mother to daughter cell. HGT has occurred frequently in all domains of life, especially in prokaryotes (such as bacteria), and contributed substantially to prokaryotic genome evolution (Lan and Reeves 1996; Ochman et al. 2000; Gogarten et al. 2002; Boucher et al. 2003; Dagan et al. 2008; Brockhurst et al. 2011; Ginty et al. 2011; Popa et al. 2011; Treangen and Rocha 2011; Bock 2017). However, the molecular mechanisms driving these HGT evolutionary events are still not fully understood. It is conceivable that in addition to the uptake and integration of foreign DNA, the proper expression of exogenous genes is also necessary for functional HGT events, which confer a selective advantage to the recipient, leading to the retention of horizontally transferred genes during evolution. However, the gene expression systems of different species are generally incompatible, and the expression elements will not function normally in a new recipient, so the freshly horizontally transferred genes are mostly silent (Thomas and Nielsen 2005; Popa and Dagan 2011). These foreign genes must be activated spontaneously and rapidly before they are lost during evolution. Promoter capture and gene duplication has been suggested to represent a major mechanism driving the activation and optimization of previously silent genes (Stegemann and Bock 2006; Matus-Garcia et al. 2012; Elliott et al. 2013; Katju and Bergthorsson 2013; Oren et al. 2014), such as the activation of citrate transporter gene in the famous *E. coli* long-term evolution experiment (Blount et al. 2012). Promoter capture is usually caused by fusion of silent gene coding region and adjacent promoter, which is mediated by DNA deletion or tandem duplication, such as the activation of histidine genes in *Salmonella typhimurium* (Anderson and Roth 1978). Our previous experimental evolution study showed that spontaneous promoter capture events prior to selection lead to rapid activation of horizontally transferred genes in *E.coli* (Li and Bock 2019). Some promoter capture events were also attributed to the evolutionary events of tandem DNA duplication, which were not mediated by classical homologous regions, and do not need any homologous sequences (illegitimate recombination). Therefore, we found that spontaneous and quick tandem DNA duplication events prior to selection pressure resulted in promoter capture, leading in turn to the rapid activation of horizontally transferred gene. Thus, rapid functional activation of a horizontally transferred gene may be mediated by quick spontaneous tandem DNA duplication. However, the regulatory mechanisms underlying this type of tandem duplication were still not clear, because this construction did not contain any reported functional elements of homologous recombination and DNA amplification. In this study, we took advantage of the aforementioned HTG activation by tandem duplication to develop a novel and simple technique for bacterial multi-copy tandem gene duplication, named tandem gene duplication selected by activation of horizontally transferred gene (TDAH), in which the tandem duplication is selected by the activation of horizontally transferred selectable marker gene.

Tandem gene duplication is a focus of research on genome evolution and is widely used in gene expression regulation and biosynthetic pathway optimization. Accordingly, a powerful technique for tandem gene duplication is very important for basic and synthetic biology. At present, there are two strategies for multi-copy tandem gene duplication, based either on in vitro or in vivo construction. In vitro methods mainly include the isocaudamer method, asymmetric sticky end complementary ligation, PCR tandem amplification, and directional adapter method (Li et al. 2003; Rao et al. 2005; Tian et al. 2007; Wu et al. 2010).Most of the existing in vitro methods used PCR amplification systems, combined with DNA restriction endonucleases and DNA ligase to artificially add repeating units and gradually increase the copy number, so that subsequent multiple transformations generate *E. coli* transformants with different copy numbers. However, these in vitro methods are often time-consuming and it is sometimes difficult to select suitable restriction enzymes, and excessive-copy tandem DNA is usually not stable in vitro. An in vivo strategy could solve the problems of in vitro methods and showed the tremendous potential for the applications in gene expression regulation and biosynthetic pathway optimization. Several in vivo methods have been reported, such as Cre/loxP-mediated multi-copy integration, amplification of a haploinsufficient gene (HapAmp), chemically inducible chromosomal evolution (CIChE), and evolution by amplification and synthetic biology (EASy) (Tyo et al. 2009; Yin et al. 2015; Peng et al. 2022; Pardo et al. 2023). In these in vivo methods, the copy numbers were selected based on the dosage of a fitness-associated gene (antibiotic resistance gene) in the repeating unit, but the mechanisms of DNA recombination and amplification were not the same and the required elements include DNA recombinases (Cre, RecA, etc.), specific DNA sequence site (*loxP*), autonomous replicating sequence (ARS), and the homologous regions, so the exogenous functional elements need to be simultaneously or beforehand introduced into the host cell; for example, EASy is mediated by synthetic DNA bridging fragment (SBF), which is an approximate 2000-bp synthetic homologous sequence and is individually transformed into host cell for homologous-recombination-mediated gene duplication. At the same time, to make the high copy number stable, the endogenous recombination system usually needs to be deleted after gene amplification to prevent subsequent homologous recombination that causes the copy number to decline. In this study, we developed a simple, stable technique for tandem gene duplication based on our previous finding that spontaneous tandem DNA duplication resulted in rapid HTG activation. TDAH construction only contains an essential selectable marker gene split at the flanks of the gene of interest, resulting in a very streamlined protocol. Moreover, TDAH worked well for both tandem DNA duplication on the *E. coli* chromosome or on a plasmid, and the copy number could be relatively stable needless of subsequent *recA* knockout. In addition to Gram-negative *E. coli*, TDAH also worked in Gram-positive *Bacillus subtilis*, indicating that it may be widely applicable in different bacteria.

As an essential micronutrient for human and animal growth and reproduction, vitamin B_2_ (riboflavin) plays crucial roles in the synthesis of flavin adenine dinucleotide (FAD) and flavin mononucleotide (FMN), as well as in the conversion of tryptophan into niacin (Averianova et al. 2020). However, the human body’s capacity for storing vitamin B_2_ is limited, leading to a relatively common occurrence of latent riboflavin deficiency, particularly among women and adolescents in developing countries (Bacher et al. 2000). Recent advancements in microbial cell factory technology have shown promise in enhancing vitamin B_2_ production. As reported, proper and reasonable expression of *ribA* was regarded as a key factor for vitamin B_2_ production (Lin et al.^2014^). We applied TDAH to select the optimal high copy number of *ribA* for vitamin B_2_ production in *E. coli*.

## Materials and methods

### Construction of TDAH starting strains

**Fig. 1:**
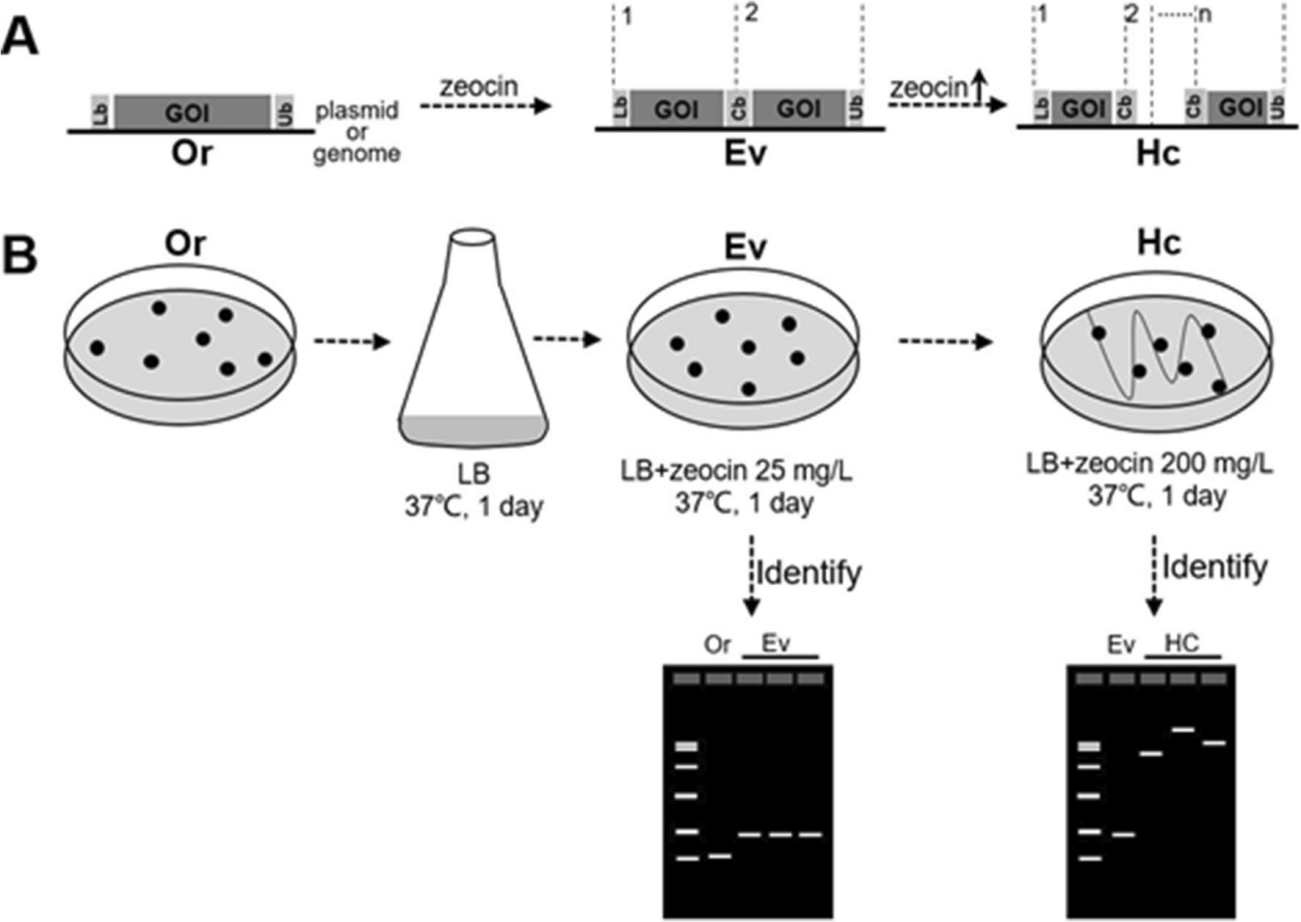
Schematic diagram of tandem gene duplication selected by activation of horizontally transferred gene (TDAH). **A** Overview of the molecular rearrangements for TDAH evolution events. In the original strain (Or), the antibiotic resistance marker *ble* expression cassette on the plasmid or the genome was split in half, the upper half of the *ble* cassette (Ub) contained promoter and was placed downstream of the gene of interest (GOI), while the lower half of the *ble* cassette (Lb) was placed upstream, so *ble* was inactive in Or. In the evolved strain (Ev), the TDAH evolution event resulted in the reconstitution of a functional *ble* expression cassette (Cb), which could be selected using antibiotics. The high-copy strain (Hc) could be selected by increasing the antibiotic concentration. **B** Experimental protocol of TDAH. The Ev strains were selected from the Or culture using by 25 mg/L zeocin, and Hc strains were selected by streaking Ev onto a plate with 200 mg/L zeocin. Ev and Hc were identified by PCR or restriction analysis

**Fig. 2:**
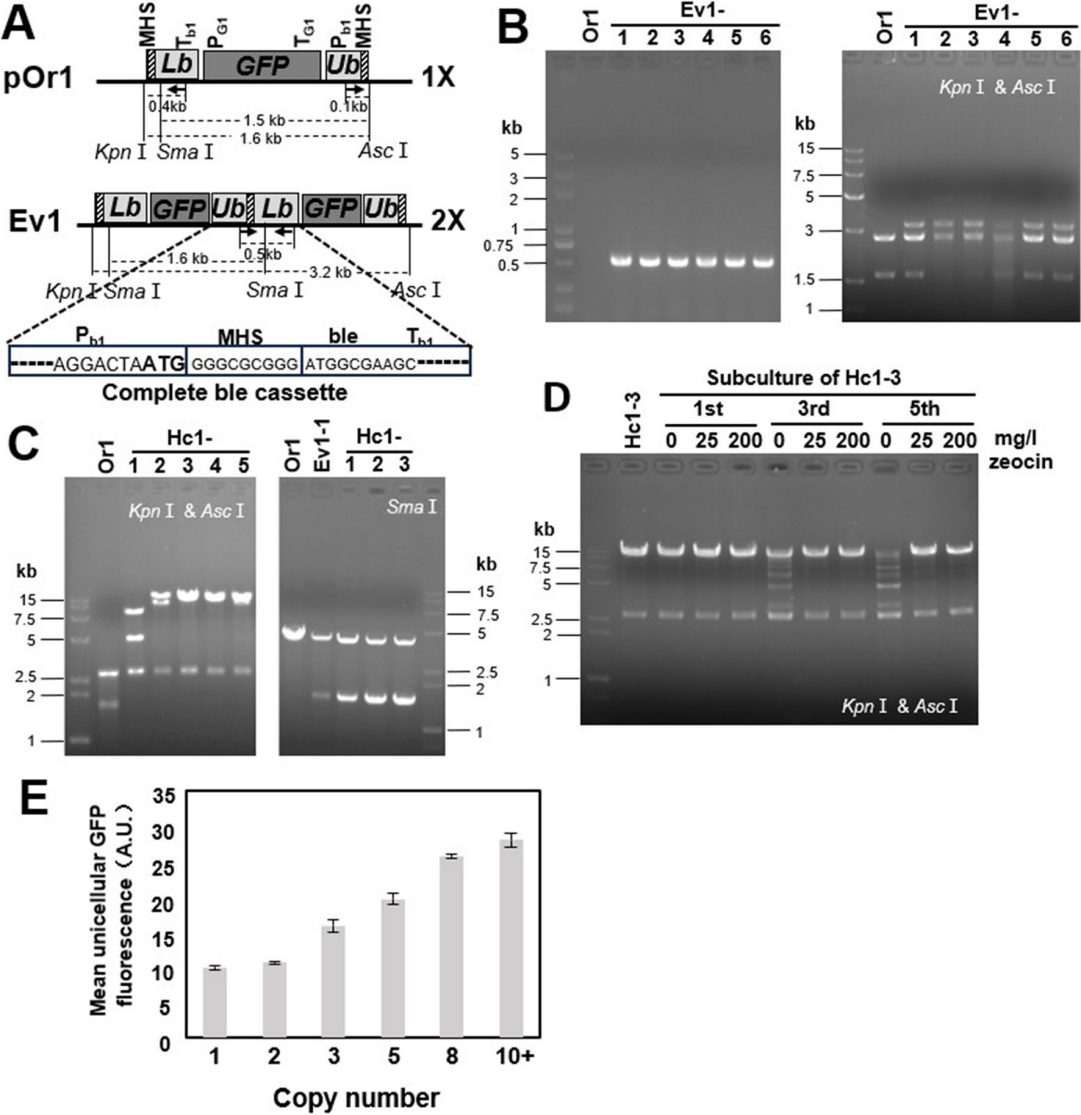
Selected and stable multi-copy tandem duplication of *E.coli* plasmid DNA using the TDAH technique and its application in gene expression regulation. **A** Schematic map of TDAH vector pOr1 and its tandem-duplication evolved plasmid (Ev1). The total length of the pOr1 vector was 4.3 kb. The length of the repeating unit was 1.6 kb, and the restriction sites (*Kpn*I and *Asc*I) are located outside of the repeating unit. *Sma*I cuts pOr1 only once (in the Lb region). Arrows indicate the primers (ble-rp and ble-fp). One 0.5-kb (0.4 + 0.1) PCR fragment obtained using the above primers indicated that the complete ble cassette (Cb) was reconstituted, and tandem duplication occurred. The *ble* gene promoter (P_b1_) and its matching terminator (T_b1_) are derived from the weak expression system J23116. Microhomology border sequences (MHS) were added flanking the repeating unit. Complete *ble* cassette in Ev1 showed the sequence information of MHS and join point; the enlarged ATG indicated the initiation codon. **B** Identification of the tandem-duplication evolved strain (Ev1). Ev1 was selected by zeocin and identified by PCR with ble-fp and ble-rp (one 0.5-kb fragment in the left electrophoretogram). To confirm the expected recombination of plasmid sequence in Ev1, Ev1 plasmids were cut with *Kpn*I and *Asc*I. In the original strain (Or), pOr1 was cut into two fragments, the backbone (2.7 kb) and one repeating unit (1.6 kb), while in Ev1, a tandem-duplication fragment appeared (1.6 × 2 = 3.2 kb in the right electrophoretogram). There was still the one-repeating-unit fragment in some Ev1 strains, indicating tandem-duplication plasmid has not been completely homogenized. **C** Identification of the high-copy strain (Hc1). The high-copy strain could be selected by higher concentrations of zeocin and was identified by the total length of the tandem-duplication region (fragment flanked by *Kpn*I and *Asc*I restriction sites). The left electrophoretogram shows that most high-copy strains (Hc1) have long restriction fragments (> 15 kb). To confirm the expected recombination of the tandem-duplication region (long restricted fragments) in Hc1, the plasmids were digested with *Sma*I, which cut once in the repeating unit, as shown in the right electrophoretogram. **D** Stability of tandem-duplication copy number of Hc1 during continuous subculture. High-copy strain Hc1-3 was subcultured in liquid LB medium for five consecutive rounds with or without antibiotic selection pressure (zeocin: 0 mg/L, 25 mg/L, and 200 mg/L). The plasmids from 1st, 3rd, and 5th subcultures were cut with *Kpn*I and *Asc*I. **E** Correlation between the tandem-duplication copy number and the expression of the gene of interest (*GFP*) in TDAH strains. The copy number was calculated based on the size of the tandem-duplication fragment (*Kpn*I and *Asc*I). The fluorescence intensity analysis indicated that the expression of the gene of interest was enhanced as the tandem-duplication copy number increased in TDAH strains

**Fig. 3:**
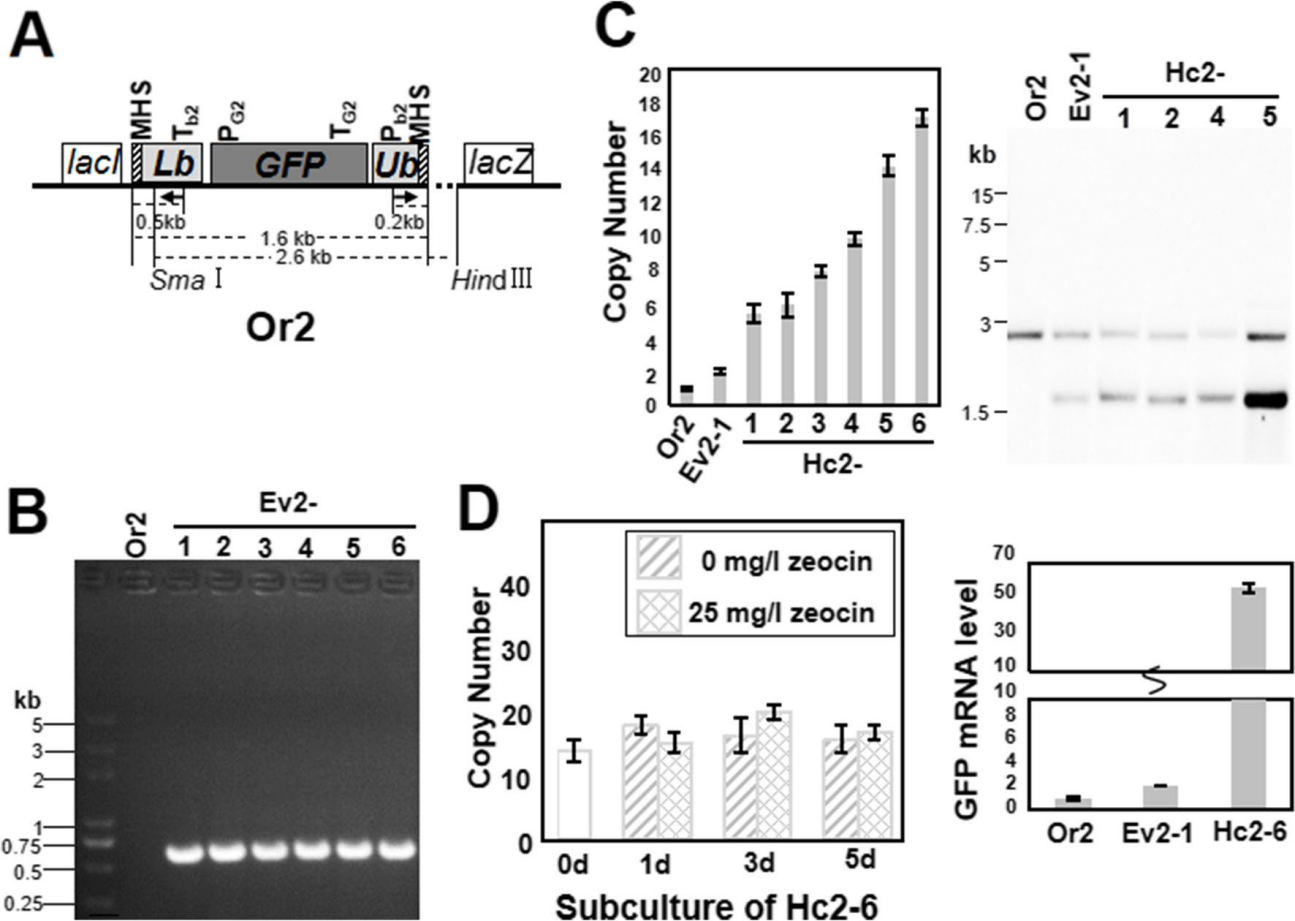
Selected and stable multi-copy tandem duplication of genome-integrated DNA in *E. coli* using the TDAH technique. **A** Schematic map of the repeating unit in the *lac* operon locus in the genome of the original strain (Or2). The length of the repeating unit was 1.6 kb. Arrows indicate primers. **B** Identification of the tandem-duplication evolved strain (Ev2). Ev2 could be selected by zeocin and identified by PCR with ble-fp and ble-rp [one 0.7 kb (0.5 + 0.2) fragment]. **C** Identification of the high-copy strain (Hc2). Hc2 could be selected by a higher concentration of zeocin. The tandem-duplication copy numbers were measured by qPCR of *ble* on genomic DNA normalized to Or2. The genomic DNA of the Hc2 strains was digested with *Sma*I and *Hin*dIII, and the Southern blot (right picture) was hybridized with a *ble* probe. **D** Stability of copy number and expression of the gene of interest in Hc2-6. Strain Hc2-6 with the highest copy number was subcultured for five consecutive rounds with or without antibiotic selection pressure (zeocin: 0 mg/L and 25 mg/L). The copy numbers were measured in the 1st, 3rd, and 5th subcultures. We did not find the obvious changes in the copy number with or without zeocin in the left histogram, indicating that the copy number remained stable during continuous subculture. As shown in the right histogram, quantification of *GFP* mRNA accumulation by qRT-PCR indicated that the high-copy strain had higher expression of the gene of interest

The schematic diagram of TDAH is shown in Fig. 1. One repeating unit as described in Fig. 2A was synthesized by GENEWIZ company (https://www.genewiz.com.cn) and was inserted into the vector backbone of pUC57, resulting in the pOr1 plasmid. The repeating unit of TDAH was made up of the lower half of the *ble* cassette (Lb), the gene of interest (GOI), and the upper half of the *ble* cassette (Ub). In pOr1, *ble* expression was driven by the weak promoter J23116 from the Registry of Standard Biological Parts (http://parts.igem.org/Part:BBa_J23119). The J23116 promoter was located in the Ub, and Lb contained the *ble* coding sequence and J23116 terminator. The GOI of pOr1 was a *GFP* gene driven by the weak araBAD promoter (Zhao et al. 2017). The original strain (Or) was constructed by introducing the pOr1 plasmid into *E. coli* strains K-12 MG1655 and DH5ɑ, respectively named as Or1 and Or4 starting strains. The pOr1 plasmid was introduced into *E. coli* strains K-12 MG1655 and DH5ɑ, respectively resulting in the Or1 and Or4 starting strains for TDAH. Based on the repeating unit of Or1, the *ble* expression system was changed to P_rrn_ (Li and Bock 2019), and the resulting repeating unit together with a kanamycin resistance gene was inserted into the lac operon locus in the genome of MG1655 by λRed recombination to form the Or2 starting strain (Fig. 3A). Table S1 shows the 150-bp homologous sequences flanking the lac locus. The pOr3 vector was constructed for TDAH of plasmid DNA in *Bacillus subtilis*, and *ble* expression was driven by the sp110 promoter (Liu et al. 2018). The GOI of pOr3 was *mCherry*, and the resulting repeating unit was inserted into the vector backbone of pHP13 to form the pOr3 plasmid. Then, pOr3 was introduced into *Bacillus* *subtilis* strain Bs168, resulting in the Or3 starting strain for TDAH. The whole open reading frame of *recA* gene was deleted by λRed recombination, and repeating unit was introduced into *lac* operon locus (Or2) to form Or5. Based on the plasmid of pOr1, with the *GFP* gene replaced by *ribA* and driven by an artificial moderately strong promoter, PWGAN-1–4 (Wang et al.^2020^), resulting in the pOr6 plasmid, the pOr6 plasmid was introduced into *E. coli* strains K-12 MG1655 to form the Or6 starting strain. All primers are shown in Table S1.

### Selection and identification of tandem duplication in the TDAH process

To select evolved strain (Ev), cultures comprising 30-mL liquid Luria–Bertani (LB) medium were inoculated with a single colony of each Or strain and cultivated at 37 °C on a rotary shaker at 180 rpm for 1 day. Aliquots of the resulting bacterial culture were spread on agar plates (15 cm diameter) with LB medium containing 25 mg/L zeocin and incubated at 37 °C overnight to identify resistant colonies (Ev strains). The liquid culture of each Or strain could be expanded to increase the cell number of starting strain and obtain more Ev strains. High-copy strain (Hc) was selected by streaking Ev on LB plates with 200 mg/L zeocin. Ev and Hc were identified by PCR or restriction analysis using the PCR primers shown in Table S1 and restriction enzymes indicated in the text and figures, respectively.

### Restriction analysis, Southern blot, real-time quantitative PCR (qPCR), real-time quantitative reverse-transcription PCR (qRT-PCR), and fluorescence analysis

Plasmids were extracted from bacterial cultures using the TIANprep Mini Plasmid Kit (Tiangen Biotech, Beijing). The restriction enzymes included *Kpn*I and *Asc*I (New England Biolabs) and *Sma*I and *Hin*dIII (Thermo Fisher Scientific). The results of restriction were analyzed by 1% agarose gel electrophoresis.

For southern blot analysis, DNA was extracted from bacterial cultures using the TIANamp Bacterial DNA Kit (Tiangen Biotech, Beijing). Restricted bacterial DNA was separated by 1% agarose gel electrophoresis and transferred to an NX nylon membrane (GE Healthcare) by capillary blotting. For preparation of hybridization probes, the *ble* coding sequence was amplified by PCR using specific primers (Table S1), and this probe was labeled with digoxin using the PCR DIG Probe Synthesis Kit (Roche, Indianapolis, IN, USA). Hybridization was performed at 47 °C for 16 h using standard protocols. Then, the membrane was washed, exposed with chemiluminescence reagent, and recorded in a luminescence imager (Tanon 5200 Multi, China).

For qRT-PCR analysis, RNA from *E. coli* cultures was extracted using the RNA prep Pure Cell Bacteria Kit (Tiangen Biotech, Beijing), and cDNA was synthesized using M-MLV Reverse Transcriptase (Promega Biotech, Beijing), Recombinant RNasin® Ribonuclease Inhibitor (Promega Biotech, Beijing), and dNTP Mixture (Biomed, Beijing). Subsequent amplification was performed using the ChamQ Universal SYBR QPCR Master Mix (Vazyme Biotech, China) on a Real-Time PCR System (ABI 7500 FAST, Applied Biosystems, USA). The *GFP* mRNA level was quantified relative to the endogenous *GAPDH* gene. Each experiment had three independent biological replicates, and the 2^−∆∆CT^ (cycle threshold) method was used to determine relative cDNA levels. Microsoft Office Excel 2010 and IBM SPSS Statistics 19 were used to analyze the experimental data. Error bars indicate the standard error for the sample mean. All primers are listed in Table S1. For the qPCR experiment, strains of Or, Ev, and Hc were used as templates, using *bioA* (in Hc2 and Hc4) and *Spec* (in Hc3) as reference genes and *ble* as target genes. Then, analysis of copies was performed based on reference genes and target genes.

The flow cytometry instrument MoFlo™ XDP (Beckman Coulter Inc. Brea, CA, USA) was used for fluorescence analysis of strains with different copy numbers. The mean value of fluorescence detected by flow cytometry was used as an index of the expression level of each strain. The cells were diluted in PBS, and GFP fluorescence was measured with the green (488 nm) laser and 529-nm filter. The forward scatter (FSC) voltage and GFP voltage were set to 150 and 450. For each sample, 100,000 events were recorded. The results were analyzed using MoFlo XDP Summit 5.2 software (Beckman Coulter Inc., Brea, CA, USA).

The concentration of vitamin B_2_ was analyzed with the wavelengths of *λ*_ex_ 473 nm and *λ*_em_ 520 nm, respectively (Chen et al. 2006).

### Copy number stability assay of the Hc strain

The assay was based on five successive subcultures of Hc at a 1:100 dilution in LB medium with or without antibiotic (zeocin: 0 mg/L, 25 mg/L, and 100 mg/L). The 1st, 3rd, and 5th subcultures were sampled, and the extracted plasmids were identified by restriction analysis (*Kpn*I and *Asc*I).

## Results

### Selected and stable multi-copy tandem duplication of *E. coli* plasmid DNA using the TDAH technique and its application in gene expression regulation

Our previous experimental evolution study found that spontaneous and quick tandem DNA duplication events (illegitimate recombination) resulted in promoter capture, leading to the rapid activation of horizontally transferred genes. In this study, we wanted to take advantage of this finding to develop a novel technique for bacterial multi-copy tandem DNA duplication, named tandem gene duplication selected by activation of horizontally transferred gene (TDAH), in which the tandem duplication is selected by the activation of a horizontally transferred selectable marker gene. Different from the existing in vivo tandem duplication methods, there are no reported functional elements of tandem duplication or homologous recombination in this TDAH system. Instead, the TDAH system only relies on the obscure evolutionary mechanism for rapid activation of horizontally transferred genes, only containing an essential selectable marker for copy number selection and only requiring the addition of this split marker gene flanking the gene of interest. This results in a very streamlined protocol, as shown in Fig. 1. We just needed to split an antibiotic resistance gene (*ble* expression cassette) in half and place Ub (containing the promoter) downstream of GOI, as well as Lb upstream, resulting in one repeating unit. Although our previous experimental evolution study (Li and Bock 2019) showed that the microhomology sequences were not necessary for duplication mutation. In order to induce our expected precise duplication mutation, we added the microhomology border sequences (GC rich, similar as the microhomology sequences found in previous study (Li and Bock 2019)) flanking the repeating unit to form our designed join point (Fig. 2A). Then, this repeating unit was introduced into the bacterial host to form the Or for TDAH. Initially, the *ble* cassette was inactive (no expression) in Or. If the target tandem-duplication event occurred, the resulting complete *ble* expression cassette (Cb) leads to functional activation of *ble* in the Ev, which can be selected on zeocin plates. In order not to introduce the foreign vector sequence into the Cb of Ev strains, we added a stop codon (TGA) upstream of Lb, avoiding the selection of strains in which the upstream vector sequence was duplicated together with Lb. A Hc could be generated and selected by increasing the antibiotic concentration.

To develop a TDAH protocol for *E. coli* plasmid sequences, we constructed the pOr1 vector (Fig. 2A), carrying a repeating unit consisting of Lb, gene of interest (*GFP*), and Ub. To be able to detect the dosage effect of the *ble* cassette, the promoter of *ble* (P_b1_) and its matching terminator (T_b1_) were based on the weak expression system J23116 from the Registry of Standard Biological Parts. The GOI of pOr1 was a *GFP* gene driven by the weak araBAD promoter (Zhao et al. 2017). The pOr1 vector was introduced into wild-type *E. coli* MG1655 to construct Or1 for TDAH. Overnight cultures of Or1 were selected on LB agar plates containing 25 mg/L zeocin, and *ble*-activated evolved strains Ev1 (resistant colonies) were further identified by PCR with primers specific for the complete *ble* expression cassette. The restriction analysis of Ev1 plasmids also confirmed the expected recombination of the evolved plasmid (Fig. 2B). The formation of Cb was also confirmed by sequencing of PCR products; even though several bp of the vector sequence was occasionally introduced between Ub and Lb in a minority of Ev strains (not affecting the expression of *ble*), almost all of Ev strains had our designed and precise join point. By counting the number of Ev colonies and the total cell number in the Or culture, we could also calculate the mutant frequency of Ev1 (colony number of Ev/total cell number of the Or culture), and it was approximately 3.6 × 10^−11^, which meant that approximately 1.6 Ev1 colonies could be selected from 100 mL of Or1 culture (Table 1).

**Table 1:** Efficiency of tandem duplication evolution using TDAH in bacteria

Original strain	Colony number of Ev/total cell number of Or culture	Colony number of Ev/100-mL Or culture
Or1	3.6 × 10^−11^	1.6
Or2	6.3 × 10^−11^	2.7
Or3	1.3 × 10^−7^	2419
Or4	3.6 × 10^−11^	1.2
Or5	0.8 × 10^−11^	0.3

Due to the dosage effect, the high-copy strain Hc1 could be selected by increasing the zeocin concentration to 200 mg/L. The resulting Hc1 colonies were further identified by the total length of the tandem-duplication region. Restriction analysis showed that most high-copy strains (Hc1) had long restricted fragments (> 15 kb), which indicated high tandem-duplication copy numbers of more than 10. To confirm the expected recombination of the tandem-duplication region (long restricted fragments) in Hc1, the plasmids were restricted with an endonuclease that cuts once in the repeating unit. The results showed that the tandem-duplication regions (long restricted fragments) contained only the repeating units with different copy numbers (Fig. 2C). Almost all colonies that grew on plates with 200 mg/L had more than two copies of the repeating unit (multi-copy). Accordingly, 3 days was sufficient to evolve the high-copy tandem DNA duplication strain from the original single-copy strain.

To test the stability of tandem-duplication copy number in Hc1, the high-copy (10 +) strain Hc1-3 was subcultured in liquid LB medium for five consecutive rounds with or without antibiotic selection pressure. The restricted plasmids from 1st, 3rd, and 5th subcultures indicated that the copy number decreased during the continuous subculture if no zeocin was added, but the normal concentration of zeocin (25 mg/L) was sufficient to maintain a relatively stable copy number (Fig. 2D). But we could still see a little bit collapse of high-copy tandem duplication after five subculture (33 generations), so based on electrophoresis image, “intensity of high-copy-tandem-duplication band (top)/intensity of plasmid backbone band (bottom)” was used to determine relative stability of high-copy-tandem-duplication (relative to starting culture (0)) (Figure S1). This analysis showed that subculture with 33 generations still had 90% of high-copy-tandem-duplication band intensity of starting culture under normal concentration of antibiotics. To our knowledge, TDAH is the first in vivo technique for high-copy tandem duplication of plasmid DNA stabilized by normal concentration of antibiotics. Classical homology-mediated methods require a very high inducing concentration to stabilize the copy number, which is obviously not suitable for routine bacterial cultivation, but stabilization of copy number without selective agent could be achieved by subsequent knockout of endogenous *recA* (Tyo et al. 2009).

However, the copy number of Hc1 strain is bound to decline after long-term subcultivation without antibiotic selection pressure. So we extended the Hc1 to 70 generations without antibiotic selection pressure, and we found that the copy number of 70th subculture [CN(70)] was 2 and copy number of Hc1 starting culture [CN(0)] was 10 [CN(0)-CN(70)]/70 = 0.11, which means average 0.11 copies lost per cell per generation during long-term subcultivation (Table 2).

**Table 2:** Analysis of collapse rate of copy number in Hc1(plasmid) and Hc2(genome)

Strain	Generation	Copy number (CN)	Collapse rate* ([CN(0)-CN(70)]/70)
Hc1	0	10	0.04
	70	2	
Hc2	0	11	0.11
	70	8	

*Unit of collapse rate: the average number of copies lost per cell per generation

To apply TDAH to regulate gene expression, *GFP* was inserted as the GOI into the pOr1 plasmid. The fluorescence intensity analysis indicated that the GOI expression was enhanced as the tandem-duplication copy number increased in TDAH strains (Fig. 2E).

### TDAH for *E. coli* genome–integrated DNA

Transgenes that are integrated into the host genome are generally more stable. We therefore developed a TDAH protocol for *E. coli* genome–integrated DNA (Fig. 3). Construction of the repeating unit was very similar to pOr1, but to obtain sufficient expression and dosage effect of the genome-integrated selectable marker gene, *ble* expression was driven by the P_rrn_ promoter (Li and Bock 2019). The repeating unit was inserted into the *lac* operon locus of the genome to form the starting strain for TDAH, which was named Or2 (Fig. 3A). The procedures for the selection and identification of the tandem-duplication evolved strain (Ev2) and high-copy strain (Hc2) were the same as above. Quantitative analysis showed that the copy number of Ev2 was 2, as expected, while the Hc2 strains had from 6 to 17 copies. Southern blot analysis showed that the tandem-duplication regions in the Hc2 strains contained only the repeating units with different copy numbers (Fig. 3B, C). Then, the highest-copy-number strain Hc2-6 was subcultured for five consecutive rounds (approximate 33 generations) without antibiotic selection pressure, and we did not find any significant changes in the copy number (Fig. 3D). Thus, the genome-integrated high-copy DNA obtained by TDAH could remain relatively stable during continuous subculture even without antibiotic selection pressure or *recA* deletion. However, the copy number of Hc strain is bound to decline after long-term subcultivation without antibiotic selection pressure. By the same way as collapse rate analysis for Hc1, collapse rate of copy number of Hc2 was 0.04, which means average 0.04 copies lost per cell per generation during long-term subcultivation (Table 2). Average number of copies lost per cell per generation indicated that tandem duplication collapsed much slower than the plasmid per generation during long-term subcultivation. The copy number of Hc1 collapsed about threefold faster than that of Hc2. Quantitative analysis of the *GFP* mRNA levels in Hc2-6 also indicated that the high-copy strain had higher expression of the GOI (Fig. 3D). The mutant frequency of Ev2 was also calculated and was found to be approximately 6.3 × 10^−11^, which meant approximately 2.7 Ev2 colonies could be selected from 100 mL of Or2 culture (Table 1).

### TDAH also works in *Bacillus subtilis*

**Fig. 4:**
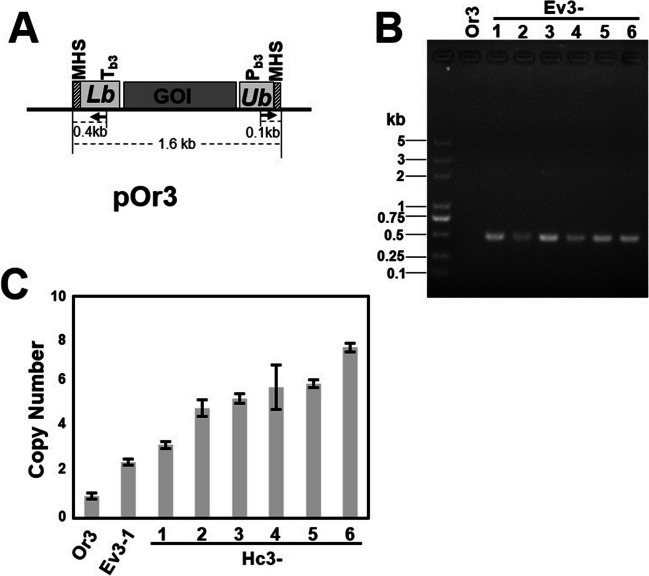
Selected multi-copy DNA tandem duplication of plasmid DNA in *Bacillus subtilis* using the TDAH technique. **A** Schematic map of the repeating unit in the TDAH vector pOr3 for tandem duplication of *Bacillus subtilis* plasmid sequences. The length of the repeating unit was 1.6 kb. Primers used for PCR and DNA sequencing are indicated by arrows. The *ble* gene promoter (P_b3_) and its matching terminator (T_b3_) were derived from the expression system sp110. **B** Identification of the tandem-duplication evolutionary strain (Ev3). Ev3 could be selected by zeocin and identified by PCR with ble-fp and ble-rp (one 0.5-kb fragment). **C** Identification of the high-copy strain (Hc3). Hc3 could be selected by higher concentrations of zeocin. Tandem-duplication copy numbers of *ble* were measured by qPCR

Since the results showed that TDAH worked well in Gram-negative *E. coli*, we also tested its effect in the Gram-positive model bacterium *Bacillus subtilis*. The pOr3 vector was constructed for TDAH of plasmid DNA in *Bacillus subtilis*. The procedures or the selection and identification of the tandem-duplication evolved strain (Ev3) and high-copy strain (Hc3) were the same as in *E. coli*. The complete *ble* cassette (Cb) could be identified in Ev3 (Fig. 4B), and higher copy numbers were selected in Hc3 strains, from 3 to 8 copies (Fig. 4C). Thus, it was demonstrated that TDAH also works in *Bacillus subtilis*. So this indicates that the promoter capture strategy and this TDAH technique for DNA tandem duplication could work well in different bacterium species. We also calculated the mutant frequency of Ev3, which was found to be approximately 1.3 × 10^−7^, which meant that approximately 2419 Ev3 colonies could be selected from 100 mL of Or3 culture (Table 1).

### *recA* deletion reduced the precision and efficiency of tandem duplication

**Fig. 5:**
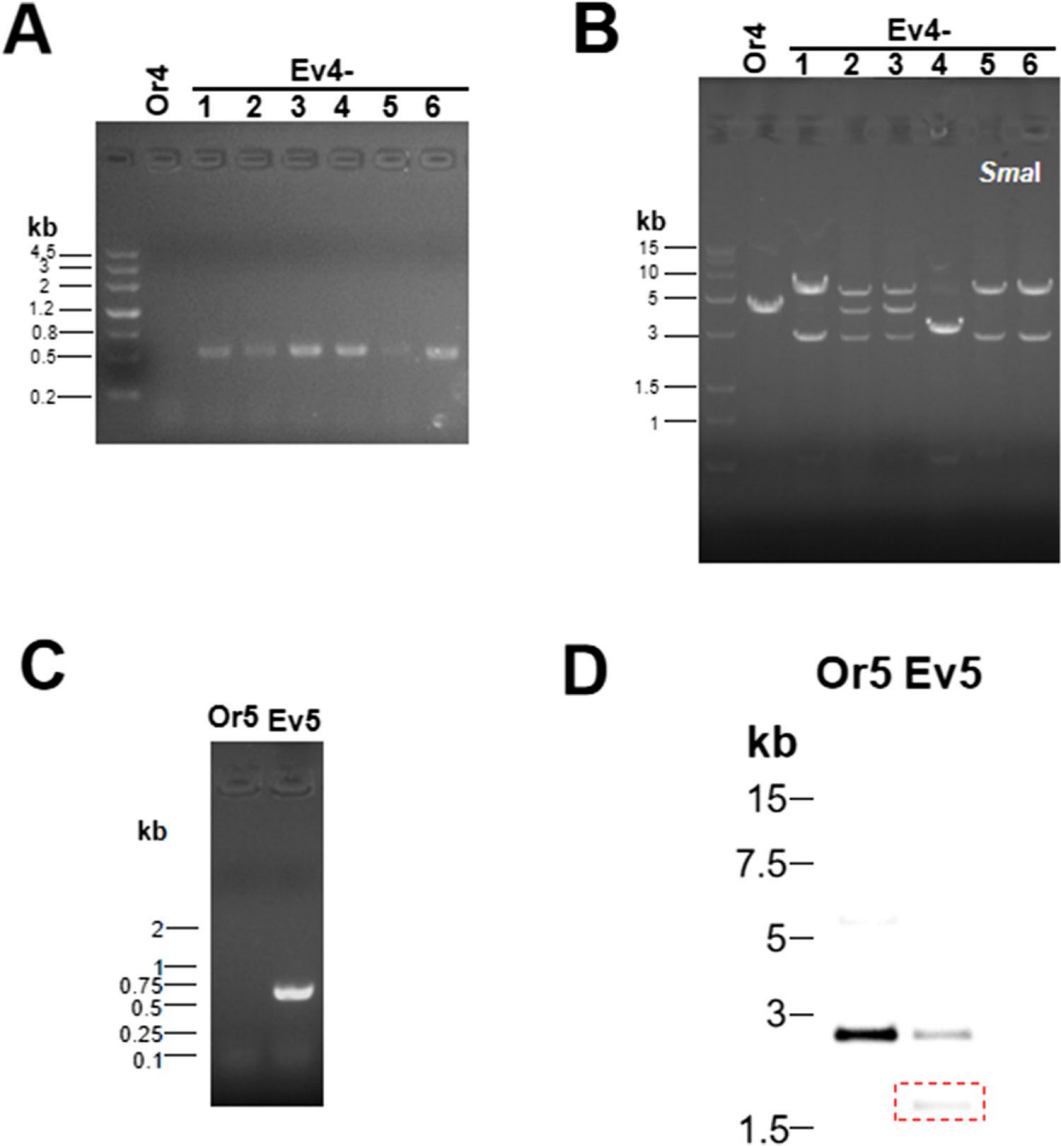
*recA* deletion reduced the precision and efficiency of tandem duplication. **A** Identification of the tandem-duplication evolved strain (Ev4) from *recA*-deficient starting strain (DH5α). The pOr1 plasmid was introduced into the *recA*-deficient *E. coli* strain DH5α, resulting in the original strain Or4. Ev4 could also be selected using the same protocol as Ev1. **B** Identification of recombinational precision for Ev4. The plasmids of Ev4 were digested with *Sma*I, which cut once in the repeating unit. The various band patterns indicate that recombinations of Ev4 take place in a chaotic manner. **C** Identification of the Ev5 from *recA*-deletion MG1655 as starting strain. DNA sequencing result indicated that the recombination of Ev5 was precise. **D** Identification of recombinational precision for Ev5. The genomic DNA of Ev5 was digested with *Sma*I and *Hin*dIII, and the Southern blot analysis was hybridized with a ble probe. Red rectangle indicates the expected 1.6-kb fragment of repeat unit

As mentioned in the introduction, different from the existing in vivo tandem duplication methods, the TDAH construction does not contain any reported functional elements of homologous recombination and DNA amplification. *recA* is an essential component required for the previously reported homologous-recombination tandem duplication methods. To investigate the role of *recA* on tandem duplication in TDAH, we chose the *recA*-deficient *E. coli* strain DH5α as the host cell for TDAH. The pOr1 plasmid (TDAH for the wild-type *E. coli* strain MG1655) was introduced into DH5α to construct the starting strain for *recA*-free, which was called Or4. And Or1 and Or4 had similar efficiency of target recombination (formation of the correct *ble* cassette) (Fig. 5A, Table 1). Next, we conducted the restriction analysis to test Ev4 (Fig. 5B), and the various band patterns indicated that recombination of Ev4 took place in a chaotic manner. The above results suggested that the knockout of *recA* reduces the precision of tandem duplication. Given that except *recA* DH5α also has several other mutations, which possibly affected duplication, we knocked out the whole open reading frame of *recA* gene from *E. coli* MG1655 and inserted the repeating unit into the lac operon locus (same as Or2) to form Or5. In order to eliminate influence of plasmid copy number in tandem duplication efficiency, we also studied the impact of *recA* on genome-integrated DNA tandem duplication efficiency. The tandem-duplication evolved strain from Or5 could also be generated using the same protocol as described for Or2, but the frequency of Ev5 significantly decreased compared to Ev2 (Table 1). DNA sequencing result indicated the recombination of Ev5 was precise (Figs. 5C). The restriction analysis also supported that the fragment arrangement of Ev5 was precise (Fig. 5D). The results above showed that *recA* was non-essential to target recombination (formation of the correct *ble* cassette) but affected the efficiency and precision of tandem duplication.

### Application of TDAH in selecting the optimal high copy number of *ribA* for vitamin B_2_ production in *E. coli*

**Fig. 6:**
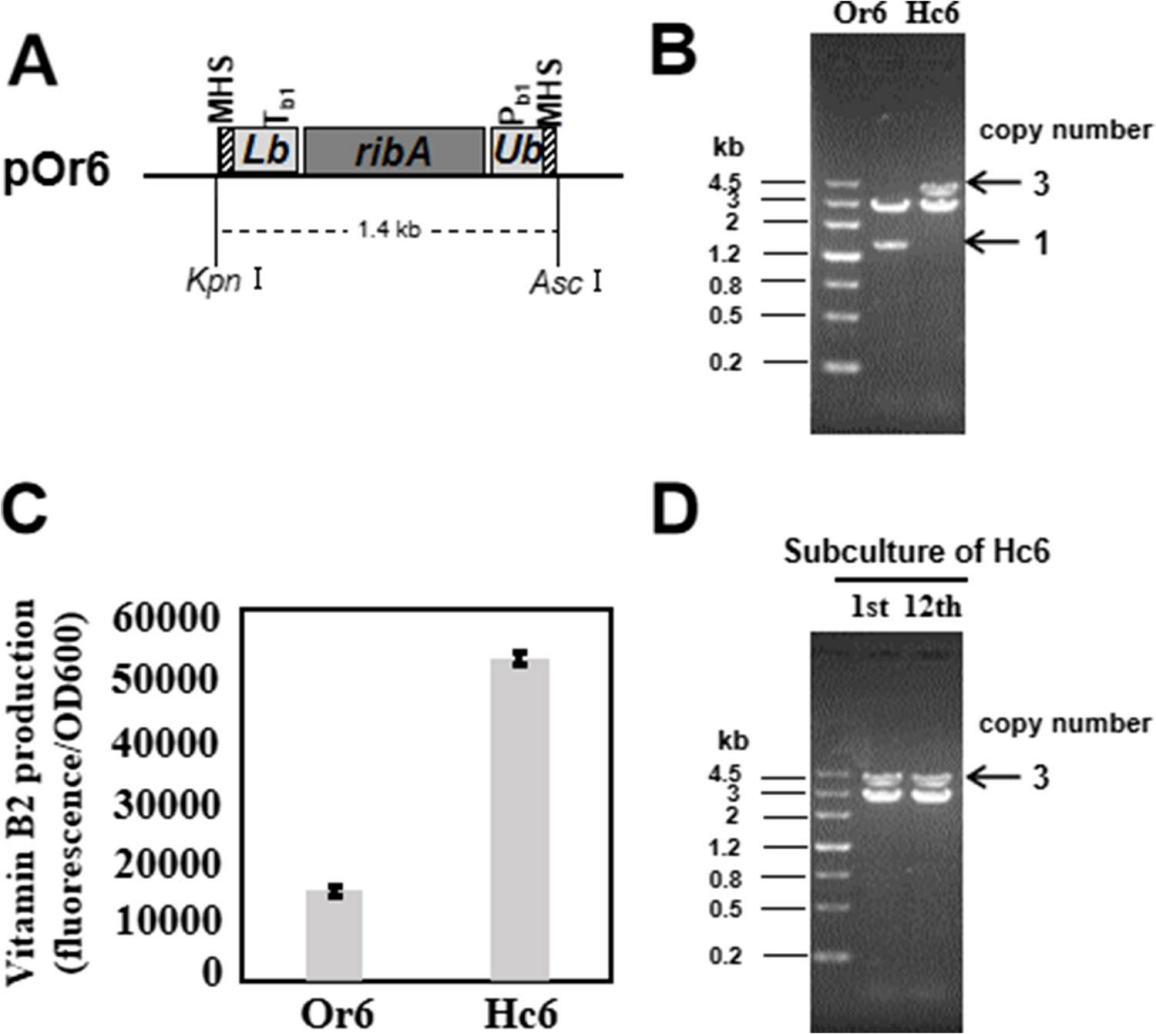
Application of TDAH in selecting the optimal high copy number of *ribA* for vitamin B_2_ production in *E. coli*. **A** Schematic map of TDAH vector pOr6. *GFP* gene of pOr1 was replaced by *ribA* gene driven by an artificial moderately strong promoter (PWGAN-1–4), producing pOr6. Total length of pOr6 is 4 kb. The length of repeating unit is 1.4 kb, and the restriction sites (*Kpn*I and *Asc*I) are located outside of the repeating unit. **B** Restriction analysis of Hc6. After several rounds of subculturing with selection of 200 mg/L zeocin, plasmid of Hc6 was restricted by *Kpn*I and *Asc*I, indicating 3 was a relatively stable copy number of *ribA* under this culture condition. **C** Vitamin B_2_ production of Or6 (single copy) and Hc6 (3 copies). **D** Restriction analysis for stability of copy number of Hc6. Hc6 was subcultured for 12 rounds with normal zeocin pressure (25 mg/L). The plasmids from Hc6 and its 12th subculture were restricted by *Kpn*I and *Asc*I

We also applied TDAH to select the optimal high copy number of *ribA* for vitamin B_2_ production in *E. coli* (Fig. 6A). High copy number of *ribA* (Hc6) was selected from single-copy starting strain (Or6). The copy number of Hc6 selected from 200 mg/L zeocin could reach up to 6 (Figure S2A), but after several rounds of subculture, the copy number would decline rapidly until stabilizing at 3 even still with selection of 200 mg/L zeocin (Fig. 6B), Under the same circumstance of cultivation, Hc6 initially selected is more yellow than its subculture, indicating more vitamin B_2_ synthesized in higher-copy Hc6 strain than in 3-copy Hc6 strain (Figure S2B). However, *E. coli* with high-copy *ribA* is not stable in current culture condition, which may be due to adverse effect of excessive *ribA* on *E. coli* growth. Three-copy Hc6 strain was relatively stable and could generate vitamin B_2_ 3.5-fold more than single-copy starting strain (Fig. 6C, D).

## Discussion

In this study, we developed a simple, fast, and stable technique for tandem gene duplication based on our previous finding that spontaneous tandem DNA duplication resulted in rapid HTG activation. The TDAH construction only contains an essential selectable marker gene split at the flanks of the gene of interest, resulting in a very streamlined protocol, and the copy number could be relatively stable needless of subsequent *recA* knockout.

Tandem gene duplication is a focus of research on genome evolution and is widely used in gene expression regulation and biosynthetic pathway optimization. Thus, a powerful technique for tandem gene duplication is very important for basic and synthetic biology. Compared with the existing in vivo tandem duplication methods, high-copy tandem-duplicated plasmid DNA obtained by TDAH could be stabilized by normal concentration of antibiotics. Classical homology-mediated or Cre/loxP-mediated methods need a very high inducing concentration to stabilize the copy number, which is obviously not suitable for routine bacterial cultivation. To our knowledge, TDAH is the first in vivo technique for high-copy tandem duplication of plasmid DNA stabilized by normal concentrations of antibiotics. The copy number of the plasmid per cell could be very high, so the stable multi-copy tandem duplication for plasmid DNA will make the copy number of plasmid-carried GOIs extremely high. In this study, we used high-copy pUC57 as the backbone for pOr1, implying that its copy number per *E. coli* cell should be approximately 600. As there were more than 10 tandem-duplicated copies of the GOI per plasmid in Hc1 (Fig. 2C), the total number of expression cassettes can reach approximately 6000 per cell. In addition, TDAH was also successfully used to obtain the table high-copy tandem duplication of genome-integrated DNA in *E. coli*. The highest copy number from one round of high-concentration-antibiotic selection was approximately 17, and the corresponding total length of the tandem-duplication region was approximately 27 kb (1.6 × 17). We believe that this is still not the upper limit of the TDAH protocol. A higher copy number should be able to be obtained by more rounds of selection or higher inducing concentrations. The genome-integrated high-copy DNA obtained by TDAH remained stable even without antibiotic pressure. However, *recA* affected the efficiency and accuracy of tandem duplication. By contrast, the existing in vivo tandem duplication methods usually need the deletion of the endogenous recombination system to stabilize the high copy number. To our knowledge, TDAH should be the first reported stable and selected system which is needless of subsequent deletion of recombination system.

The reason why the high-copy tandem duplications are more stable than those obtained by other in vivo methods may be that TDAH uses a different and obscure molecular mechanism of tandem DNA duplication, which is supported by the fact that there are no reported DNA recombination and amplification elements in the TDAH construction. Thus, non-homologous mediates the tandem-duplication in the TDAH system. Researchers have found that many naturally occurring bacterial DNA duplication events were not mediated by sequence identity (Reams and Neidle 2004; Brochet et al. 2008; Reams et al. 2010; Lin et al. 2011; Sun et al. 2012; Elliott et al. 2013), indicating the importance of illegitimate recombination in bacterial DNA duplication. However, the evolutionary benefits and mechanisms of these illegitimate recombination events are still incompletely understood. Our previous study (Li and Bock 2019) and this work indicate that one of the evolutionary benefits would be the activation of previously silent horizontally transferred genes and the optimization of their expression. Nevertheless, bacterial illegitimate recombination is highly complex and still somewhat obscure. Researchers have found that many Rec proteins (RecE, RecF, RecJ, RecO, RecR, RecQ, etc.) participate in the regulation of illegitimate recombination in bacteria (Ukita and Ikeda 1996; Hanada et al. 1997; Shimizu et al. 1997; Shiraishi et al. 2002), but it is still not clear which could regulate the illegitimate recombination in TDAH or during the rapid activation of horizontally transferred genes. To our knowledge, TDAH is the first DNA duplication technique based on illegitimate recombination, and although illegitimate recombination was considered to be inefficient, uncontrollable, and unstable, TDAH offers high efficiency and stability. Because the TDAH construction does not contain any functional elements used in other in vivo methods and is based on a different mechanism, joint use of TDAH and other in vivo methods may further improve the efficiency of tandem duplication. This would require the addition of another selectable marker gene (split) flanking the original repeating unit, which will not affect the original tandem duplication system and is easy to construct. However, the combination of TDAH with other methods is beyond the scope of this paper and should be investigated in future studies.

The promoter capture strategy and this TDAH technique for DNA tandem duplication TDAH should be applicable in most laboratory strains and industrial hosts. We also found evidence that the molecular mechanism underlying TDAH should be conserved among bacteria, as it worked well both in Gram-negative *E. coli* and Gram-positive *Bacillus subtilis*. This indicates that the application of TDAH cloud also be extended to other organisms, such as yeasts, fungi, and even mammalian cell lines. This is easy to test, because TDAH is simple and fast, and the only technical requirement is a genetic transformation method and selection system. Interestingly, we found the efficiency of tandem duplication evolution using TDAH in *B. subtilis* was four orders of magnitude higher than that of *E. coli*. We speculated that this may be due to the differences in the mutation rate or the regulation details of illegitimate recombination between these two species. One important application of the tandem DNA duplication technique is the regulation of gene expression, which is widely used in recombinant protein production and expression optimization of biosynthetic pathways (Tyo et al. 2009; Yin et al. 2015; Peng et al. 2022). In this study, TDAH also showed a powerful ability to regulate gene expression. As expected, GOI expression was enhanced as the tandem-duplication copy number increased in TDAH strains (Figs. 2E and 3D). The scope of copy number variation was rather wide (Figs. 2C and 3C) and can be further tuned by subcultivation of plasmid-carried high-copy tandem-duplicated strains without the selection pressure (Fig. 2D). Thus, TDAH can potentially also be used to develop a library of strains with varying expression levels for one gene or multi-gene combinations. TDAH is therefore expected to become a powerful tool for in vivo tandem duplication of plasmid-carried and genome-integrated genes that is selected, stable, widely applicable, simple, and fast.

During the selection of the optimal high copy number of *ribA* for vitamin B_2_ production. *ribA* copy number initially reached to 6 and then rapidly declined until stabilizing at 3. Although a positive correlation was observed between *ribA* copy number and vitamin B_2_ biosynthesis, *E. coli* with high-copy *ribA* is not stable in current culture condition, which may be due to adverse effect of excessive *ribA* on *E. coli* growth. As reported, NADH is essential for vitamin B_2_ biosynthesis (Abbas and Sibirny 2011, Liu et al. 2021), but NADH generation pathway was not improved corresponsively in Hc6. The imbalance between the large biosynthesis flux of vitamin B_2_ and the intracellular limited NADH adversely affected cell growth and resulted in a decrease in *ribA* copy number. Initial high copy number could be fixed by *recA* deletion, but this may not lead to ultimate vitamin B_2_ production of *E. coli* culture improved and stable; we speculate that vitamin B_2_ biosynthesis driven by 3-copy *ribA* is maximal biosynthesis flux, which intracellular NADH could support in current culture condition; thereby, 3 is the optimal copy number of *ribA* for vitamin B_2_ production in our experiment.

## Supplementary Information

Below is the link to the electronic supplementary material.

Supplementary file1 (PDF 252 KB)

## Data Availability

All data generated or analyzed during this study are included in this published article and its supplementary information files.
