# Supplementary material for: Tandem gene duplication selected by activation of horizontally transferred gene in bacteria
Source: Appl Microbiol Biotechnol. 2024 May 23;108(1):340. doi: 10.1007/s00253-024-13160-z (PMC11111574; doi:10.1007/s00253-024-13160-z)
Supplement: Supplementary file 1 — Supplementary file1 (PDF 252 KB) [file 253_2024_13160_MOESM1_ESM.pdf]

Applied Microbiology and Biotechnology

Tandem gene duplication selected by activation of horizontally transferred gene in bacteria

Fangqing Zhang[1,2,3,†], Xinxin Shi[1,2,†], Jian Xu[1,2], Wen Yuan[1,2]&ZhichaoLi[1,2,*]

[1] Key Laboratory of Systems Microbial Biotechnology, Tianjin Institute of Industrial Biotechnology, Chinese Academy of Sciences, Tianjin 300308, China.
[2] National Technology Innovation Center of Synthetic Biology, Tianjin 300308, China.
[3] Key Lab of Industrial Fermentation Microbiology of the Ministry of Education, School of Biotechnology, Tianjin University of Science and Technology, Tianjin, China

[*]: Corresponding author: Zhichao Li, e-mail: (lizhch@tib.cas.cn), Tianjin Institute of Industrial Biotechnology, Chinese Academy of Sciences, Tianjin 300308, China. phone: +8602224828727

[†]: Fangqing Zhang and Xinxin Shi shared equal contributions to the paper and should be considered co-first authors.

Supplementary figures

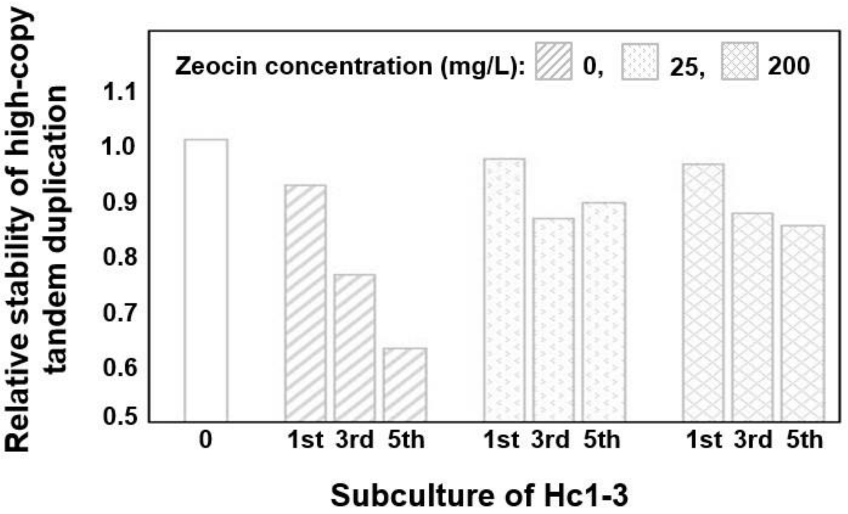

Figure S1 Stability of tandem-duplication copy number of Hc1 during the continuous subculture. Based on Figure 2D, "intensity of high-copy-tandem-duplication band (top) / intensity of plasmid backbone band (bottom)" was used to determine relative stability of high-copy-tandem-duplication [relative to starting culture (0)]. The intensity of visualized band was quantified by software ImageJ.

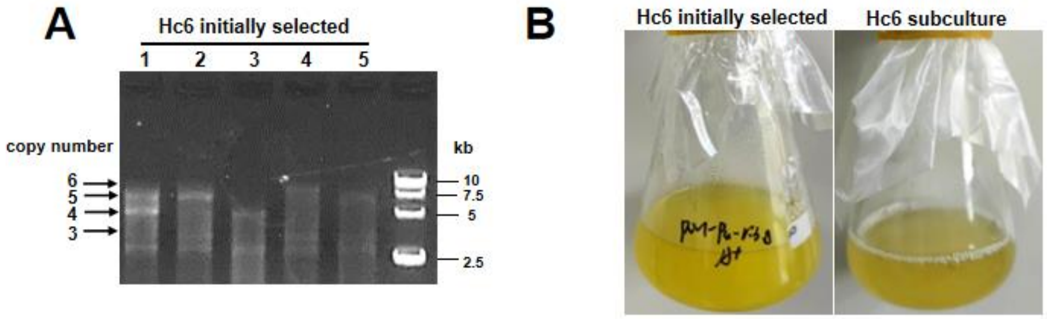

Figure S2 Hc6 initially selected had higher copy number of *ribA* and synthesized more vitamin B2. (A) Restriction analysis of Hc6 initially selected from 200 mg/L zeocin. Plasmid of Hc6 initially selected from 200 mg/L zeocin was restricted by *Kpn*I and *Asc*I, indicating the copy number of Hc6 initially selected could reach up to 6. (B) Comparison between Hc6 initially selected and its subculture. Under the same circumstance of cultivation, Hc6 initially selected is more yellow than its subculture, indicating vitamin B2 biosynthesis of higher-copy Hc6 strain is more than that of 3-copies Hc6 strain.

Supplementary tables

Table S1 List of oligonucleotides used in this study.

Primers for the vector construction

Target sequence	Forward primer (5′-3′)	Reverse primer (5′-3′)
Prrn promoter	GCTCCCCCGCCGTCGTTCAA	ATGTATATCTCCTTCTTAAA
lac operon left flanking sequence	AGAAAAACCACCCTGGCGCC	GGTGCCTAATGAGTGAGCTA
lac operon right flanking sequence	GTTTCTTTATGGCAGGGTGA	ATTCGGGATTTCGGCGCTCC
sp110promoter	GAAAATATCAAAAATGTGTT	CTCCAATTATATCATACATT

Primers for the identification of Ev strains

Target sequence	Forward primer (5′-3′)	Reverse primer (5′-3′)
Ev1	AGTCCTAGGGACTATGCTAGC	ACTCTAGTAGAGAGCGTTCACC
Ev2	ATACCGGCTCCCCCGCCGTCG	ACTCTAGTAGAGAGCGTTCACC
Ev3	GAAAATATCAAAAATGTGTTG	AAAAAAAACGCCCGGCTTTCAC

Primers for the generation of hybridization probes

Gene	Forward primer (5′-3′)	Reverse primer (5′-3′)
Ble	AGGACGACTTCGCCGGTGTG	CGGCTGCTCGCCGATCTCGGT

Primers for real-time qPCR and qRT-PCR.

Gene	Forward primer (5′-3′)	Reverse primer (5′-3′)
Ble	AGGACGACTTCGCCGGTGTG	CGGCTGCTCGCCGATCTCGGT
Spec	ATGTTTGGATCAGGAGTTGA	CCACGGTACCATTTCTTGCT
bioA	GTGATGCCGAAATGGTTGCC	GCGGTCAGACGCTGCAACTG
GFP	AGCTGACCCTGAAGTTCATCTG	TGTAGTTGTACTCCAGCTTGTG
GAPDH	GAAAGCGAAAGGCGCAGAAA	TTGCCATCCAGAGTGTCGTC
